# The Surgical Intervention for Traumatic Injury Scale: A Clinical Tool for Traumatic Brain Injury

**DOI:** 10.5811/westjem.2019.4.41802

**Published:** 2019-06-18

**Authors:** Eric A. Sribnick, Michael Lunney, David W. Wright, Jason W. Allen, Patricia A. Hudgins, Junxin Shi, Krista Wheeler, Jeffrey R. Leonard, Sanjay S. Dhall, Henry Xiangh

**Affiliations:** * Nationwide Children’s Hospital, Division of Neurosurgery, Columbus, Ohio; † The Ohio State University, Department of Neurosurgery, Columbus, Ohio; ‡ Nationwide Children’s Hospital, Center for Pediatric Trauma Research, Columbus, Ohio; § Emory University, Department of Emergency Medicine, Atlanta, Georgia; ¶ Emory University, Department of Radiology and Imaging Sciences, Atlanta, Georgia; || University of California, San Francisco, Department of Neurosurgery, San Francisco, California

## Abstract

**Introduction:**

There is no widely used method for communicating the possible need for surgical intervention in patients with traumatic brain injury (TBI). This study describes a scoring system designed to communicate the potential need for surgical decompression in TBI patients. The scoring system, named the Surgical Intervention for Traumatic Injury (SITI), was designed to be objective and easy to use.

**Methods:**

The SITI scale uses radiographic and clinical findings, including the Glasgow Coma Scale Score, pupil examination, and findings noted on computed tomography. To examine the scale, we used the patient database for the Progesterone for the Treatment of Traumatic Brain Injury III (ProTECT III) trial, and retrospectively applied the SITI scale to these patients.

**Results:**

Of the 871 patients reviewed, 164 (18.8%) underwent craniotomy or craniectomy, and 707 (81.2%) were treated nonoperatively. The mean SITI score was 5.1 for patients who underwent surgery and 2.5 for patients treated nonoperatively (P<0.001). The area under the receiver operating characteristic curve was 0.887.

**Conclusion:**

The SITI scale was designed to be a simple, objective, clinical decision tool regarding the potential need for surgical decompression after TBI. Application of the SITI scale to the ProTECT III database demonstrated that a score of 3 or more was well associated with a perceived need for surgical decompression. These results further demonstrate the potential utility of the SITI scale in clinical practice.

## INTRODUCTION

In the United States, traumatic brain injury (TBI) leads to significant morbidity and mortality with recent data showing that patients reaching a hospital with TBI account for more than 250,000 hospital admissions and more than 50,000 deaths.^1^ While there are no approved pharmacotherapeutic agents for the treatment of TBI, timely management at an appropriate institution may improve outcomes.^2^ One method for potentially facilitating communication and management of this patient population is the use of a clinical decision tool.

When designed and used appropriately, clinical decision tools have been shown to improve clinical practice.^3^ Currently, there are no widespread clinical decision tools for the evaluation and surgical treatment of TBI. The Glasgow Coma Scale (GCS) has been extensively used to classify TBI patients by injury severity and is a well-defined and reproducible system;^4^ however, this scale does not provide information to indicate whether a surgical intervention is necessary.^5^ We previously described the Surgical Intervention for Traumatic Injury (SITI) scale as a possible clinical decision tool for evaluating a patient’s potential need for surgical decompression (craniotomy or craniectomy) for treatment of TBI.^6^

Our currently presented findings expand on that original study by using the database from a recent multicenter study for TBI. The Progesterone for Traumatic Brain Injury, Experimental Clinical Treatment (ProTECT III) trial was a prospective, randomized clinical trial that examined the effect of treatment with intravenous progesterone vs placebo in patients with nonpenetrating, moderate or severe TBI.^7^ We used the emergency department (ED) admission data, head computed tomography (CT) findings at presentation, and the surgical treatment data from the ProTECT III trial to determine if the patient’s score on the SITI scale correlated with whether they received a surgical decompression within the first 24 hours of admission. Our hypothesis was that the SITI score, at the time of admission, would be significantly higher in patients who went on to have surgical decompression.

## METHODS

The ProTECT III trial met institutional requirements for the conduct of human subjects research and was registered on http://www.ClinicalTrials.gov (identifier, NCT00822900). The currently presented study used de-identified data from the ProTECT III database; nonetheless, we sought approval by the institutional review board (IRB). The IRB determined that review was not necessary.

### Patient Data

This was a retrospective study that used an existing database from the ProTECT III trial.^7^ The ProTECT III trial was a phase III, multicenter, double-blind, clinical trial examining the efficacy of progesterone for the treatment of TBI. Inclusion criteria for the ProTECT III trial were adults with blunt force TBI and an initial GCS combined score of 4–12 who were able to initiate treatment within four hours of injury. Exclusion criteria included the following: an injury deemed nonsurvivable; a clinical exam demonstrating bilateral dilated and unresponsive pupils; clinical evidence of hypoxemia, hypotension, spinal cord injury, or status epilepticus; a history of cardiopulmonary resuscitation following the injury; a current pregnancy; a history of reproductive cancer or a blood clotting disorder; a current diagnosis of active myocardial infarction, ischemic stroke, pulmonary embolism, or deep vein thrombosis; allergy to either progesterone or the pharmacological delivery vehicle; severe alcohol intoxication (defined as having an ethanol level greater than 249 milligrams per deciliter); or being a ward of the state (e.g., a prisoner). In addition, for analysis for the current study, patients were removed if they presented with intraparenchymal hemorrhage in the posterior fossa or if surgical intervention was not considered (eg, the family decided to withdraw care, or surgery was excluded as an option by the treating physicians).

**Population Health Research Capsule**

What do we already know about this issue?

The Glasgow Coma Scale is widely used to classify severity of traumatic brain injury (TBI). It does not measure potential need for surgery in patients with TBI.

What was the research question?

Does the Surgical Intervention for Traumatic Injury [SITI] scoring system correlate with the decision to perform a craniotomy for TBI?

What was the major finding of the study?

When applied to the database of ProTECT III (a clinical trial of progesterone to treat TBI), scores on the SITI scale correlated with a perceived need for craniotomy

How does this improve population health?

While our results need prospective evaluation, the SITI scale may be a clinical decision tool that can efficiently communicate potential surgical urgency in TBI patients.

We reviewed the patient report forms from the ProTECT III trial to ensure that the data collected would be sufficient for calculating the SITI score. The variables needed to determine the SITI score were mapped to the data elements from the original ProTECT III public-use data set, and a single database was created using SPSS (IBM, Armonk, New York). Specifically, the data used for this study included demographic information, mechanism of injury, timing from injury to arrival to the ED, the combined GCS score on arrival, pupillary response on arrival, data obtained from the radiologist’s interpretation of the admission head CT, and information regarding surgical interventions. All patients included and randomized to the ProTECT III study had a calculated GCS performed in the ED. For intubated patients, the verbal response was graded 1T. None of the patients included in the ProTECT III trial were found to have a history of prior eye surgery that would have prevented performance of a pupillary light reflex.

For patients with midline shifts that were not clear from the ProTECT III database, a radiologist (Jason W. Allen) blinded to the patient’s background information determined the degree of midline shift. In cases where the patient’s operative status was unclear (ie, whether the patient had an operation in the first 24 hours after admission), individual case reviews were performed to determine whether the patient received surgical intervention. We defined patients as “operative patients” if they had craniotomies or craniectomies within 24 hours of arrival to the hospital. In ProTECT III, craniectomy and craniotomy were considered third-tier therapy. Surgeons were advised to perform surgical intervention, at their discretion, for refractory intracranial pressure and were referred to the most recent surgical guidelines.^5^

### SITI Scale

The SITI scale was previously described (Table 1),^6^ and its design was influenced by published surgical guidelines.^5^ Briefly, the scale has five components: the combined GCS score on initial evaluation in the ED; eye findings; midline shift on head CT; presence of blood within or near the temporal lobe on head CT; and presence of an epidural hematoma on head CT.

To calculate the SITI score, we obtained the GCS combined score from the patient’s initial evaluation in the ED, Patients with total GCS scores of 9–12 received 1 point, and patients with total GCS scores of <9 received 2 points. On the initial eye exam, a unilateral enlarged pupil added 2 points. (Bilateral enlarged and/or unreactive pupils did not add points.) Findings on head CT were also used: we measured midline shift of the septum pellucidum (measured at the level of the foramen of Monro), and patients received 2 points for midline shift measuring 5–10 millimeters (mm) and 4 points for midline shift >10 mm. Pathology (defined as hemorrhage or edema) localized to the middle cranial fossa added 1 point. An epidural hematoma with a width ≥ 10 mm added 2 points. The minimum score was zero, and the maximum possible score was 11.

### Statistical Analysis

The statistical analyses were performed by a statistician (Junxin Shi), and the software Statistical Analysis System 9.3 (SAS Institute, Cary, North Carolina) was used. We compared operative and nonoperative patient groups using t-tests for means and chi-squared tests for percentages (statistical significance was defined as *P*<0.05). We built logistic models to examine the odds of surgery with varied combinations of the five SITI score components as independent variables. For each of these models, we constructed area under the receiver operating curves (AUC) to evaluate the SITI scale’s performance.^8^ For the final chosen model, using all five SITI score components, we report sensitivity, specificity, positive predictive value, and negative predictive value.

## RESULTS

### Characteristics of Study Subjects

**Figure 1:**
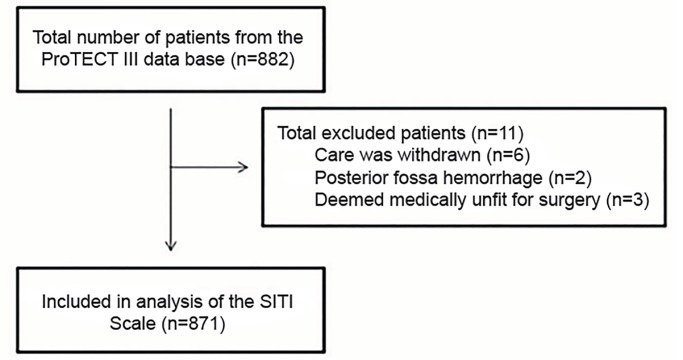
Flowchart of retrospective patient selection from the ProTECT III database. *ProTECT III*, Progesterone for the Treatment of Traumatic Brain Injury III Trial; *SITI*, Surgical Intervention for Traumatic Injury.

Of the 882 patients enrolled in the ProTECT III trial, 871 patients were assessed. Eleven patients were not assessed for this retrospective analysis: six of the patients had care withdrawn; two had a posterior fossa hemorrhage; and three were deemed medically unfit for surgery by their treating physician (Figure 1). Patient characteristics were examined by univariate analysis (Table 2). Comparing the operative and nonoperative patients, we found no difference in gender or intubation status. Operative patients were, on average, six years older than nonoperative patients (*P*<0.001), and operative patients were transported from the location where the injury took place to the admitting hospital, on average, eight minutes earlier than nonoperative patients (*P*<0.001). For the components of the SITI score, operative patients had a slightly higher GCS combined score (*P*= 0.047), a higher rate of a unilateral enlarged pupil on initial exam (*P*=0.015), and higher rates of midline shift, temporal pathology, and epidural hematoma (*P*<0.001, for each variable). Treatment with progesterone for the ProTECT study was similar between the two groups (*P*=0.82).

### Main Results

**Figure 2:**
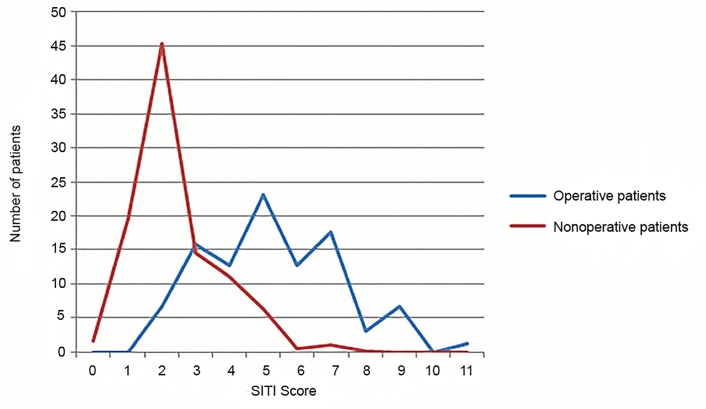
The Surgical Intervention for Traumatic Injury (SITI) score at admission for operative and nonoperative patients.

**Figure 3:**
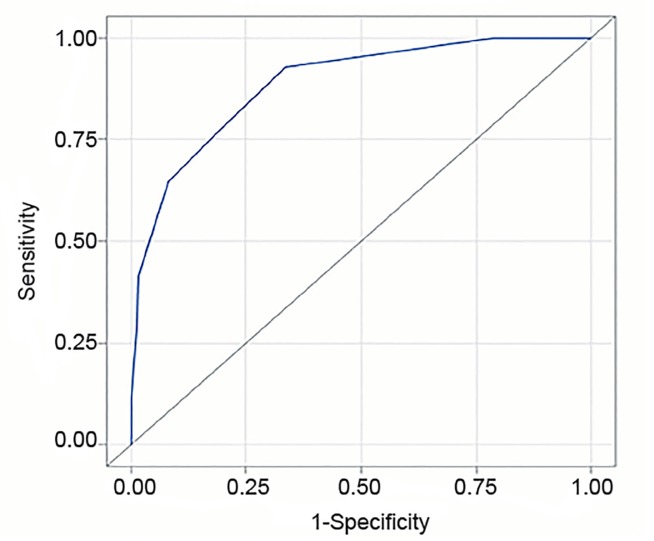
The area under the receiver operating characteristic (ROC) curve using a Surgical Intervention for Traumatic Injury (SITI) score of 3 as the threshold. Area under the curve = 0.8866.

Comparing the percentages of patients who had certain SITI scores, approximately 66.5% of the nonoperative patients had SITI scores between of 0 and 2, as compared with 6.7% of the operative patients (Figure 2). To determine the potential usefulness of setting the threshold of a positive SITI score at 3 or above, we performed retrospective analysis. The sensitivity for the SITI score with the decision to perform a craniotomy or craniectomy was 0.93, and the specificity was 0.66 (Table 3). The positive predictive value was 0.39, and the negative predictive value was 0.97 (Table 3). The AUC was also examined and was found to be 0.89 (Figure 3).

## DISCUSSION

As was shown in the initial publication describing the SITI score,^6^ our results indicate that there is a strong association between the SITI score and a neurosurgeon’s perceived need to perform a craniotomy or craniectomy for treatment of TBI. Our work represents an initial effort to create such a tool, and there is no gold standard to use for comparison. To further examine the SITI score, we used AUC analysis, which is a well-recognized method of evaluating a diagnostic test.^9^

The AUC for the SITI score was found to be 0.89, indicating that higher SITI scores were associated with patient presentations that neurosurgeons perceived as requiring surgical intervention.^10^ For comparison, in a multicenter study the commonly used Acute Physiology, Age, Chronic Health Evaluation (APACHE III) methodology was found to have an AUC of 0.89 for prediction of mortality in trauma patients admitted to the intensive care unit.^1^ In addition, the SITI score had a high sensitivity and a high negative predictive value, suggesting that it would have a higher tendency to identify patients who potentially need surgery and would have a lower tendency to mislabel potentially operative patients as nonoperative.

The clinical implications of such a scale are several-fold. A validated numerical scale could promote clear and efficient communication between clinicians in the manner similar to how the GCS is used to rapidly communicate a neurological assessment.^12^ The SITI score could be used in interdepartmental communication (e.g., between the ED and the neurosurgery consultant) or for hospital-to-hospital transfer (e.g., between a referring hospital and an accepting trauma center). Increasing efficiency in communication for patient transfers may translate into improved outcomes, as earlier operative intervention may improve functional outcome.^13^

The current study advances our research of the SITI scale as a clinical tool. Our initial retrospective study^6^ did show a possible association between the SITI score and the surgeon’s decision to perform a surgical decompression, but that study had several limitations, including that it was limited to a single-center, retrospective design, a limited number of patients, and had a high potential for observer bias. While the current study was also retrospective, the data were from a Phase III, multicenter trial where TBI patient treatment and outcome data were collected for a completely separate purpose; thus, observer bias was not likely introduced. Nonetheless, future work on the SITI scale will need to include prospective analysis.

Determining the utility of the SITI score in clinical practice will require prospective testing and, ultimately, clinician acceptance. Nonetheless, prior research has identified several aspects of a clinical decision tool that were predictive of usefulness: the SITI scoring system is automated; it provides information at the time of clinical decision making; and it provides a recommendation that can result in a clinical intervention.^3^ The SITI scoring system is based on information that should already have been gathered for the TBI patient. It would easily lend itself to a handheld device (e.g., tablet or smartphone). Additionally, the information provided by the score would arrive at the time a decision needs to be made and would support a clinical action.

For a scoring system to be effective, it must define a specific clinical scenario and population to be addressed. For instance, the commonly used Subaxial Cervical Spine Injury Classification (SLIC) is not applicable to the entire cervical spine, as injuries involving the atlas, axis, and craniocervical junction are distinct injuries that do not lend themselves to the SLIC scale.^14^ Finally, a clinical scale should be used to suggest a clinical response, not to dictate it. The over-riding point of the scale is not to replace clinical judgment but to highlight a patient population in which timely surgical action may be warranted.

## LIMITATIONS

A limitation to the SITI scale is that it is not intended to be used for all forms of TBI. It only covers closed head injury; skull fractures do not factor into the score, and it does not address posterior fossa injuries. Guidelines for the surgical management of penetrating head injury^15^ and depressed skull fracture^16^ exist and have additional considerations, such as infection prevention, that also must be taken into account when deciding on surgical management. Injuries to the posterior fossa have their own indications and are rare.^17^–^19^ The current study uses data obtained from the ProTECT III trial; thus, any exclusion criteria from that study (e.g., severe alcohol intoxication) influenced the present study and limit its applicability. Future work will need to be more inclusive to demonstrate the utility of this clinical tool.

## CONCLUSION

In summary, this study used the multicenter ProTECT III database to examine whether the previously described SITI scoring system correlates with TBI patients who received surgical intervention for their injury. Our findings show a strong association between a SITI score of 3 or greater and the treating neurosurgeon’s perceived need to perform an operative intervention. Our findings potentially have significant clinical implications. Utility of the SITI score in clinical practice and future clinician acceptance require further prospective evaluation.

## Figures and Tables

**Table 1:** Components of the Surgical Intervention for Traumatic Injury Scale.^6^

Feature	Finding	Points
GCS	>12	0
	9–12	1
	<9	2
Eyes		
Unilateral enlarged pupil	yes	2
	no	0
Head CT	<5 mm	0
Midline shift	5–10 mm	2
	>10 mm	4
Temporal blood	yes	1
	no	0
Epidural hematoma >10 mm	yes	2
	no	0

*GCS*, Glasgow Coma Scale; *CT*, computed tomography; *mm*, millimeter.

**Table 2:** Patient characteristics.

	Nonoperative	Operative
Total number of patients, n	707	164
Mean age, years‡	37.8	44.2
Female patients, n^a^	184 (26.0)	44 (26.8)
Mechanism, n^a^‡		
MVC/ATV/Scooter	425 (60.1)	62 (37.8)
Fall	101 (14.3)	33 (20.1)
Assault	38 (5.4)	16 (9.8)
Bicycle	38 (5.4)	8 (4.9)
Other/unknown	23 (3.2)	13 (7.9)
Pedestrian struck by vehicle	82 (11.6)	32 (19.5)
Mean time from injury to ED intake (minutes)‡	55.1	47.2
Intubation, n^a^‡	169 (23.9)	41 (25)
Mean GCS†	7.6	8.1
Enlarged pupul, n^a^‡	94 (13.2)	34 (20.7)
Midline shift, n^a^‡		
0–5 millimeters	688 (97.3)	61 (37.2)
5–10 millimeters	19 (2.7)	68 (41.5)
> 10 millimeters	0	35 (21.3)
Temporal pathology, n^a^‡	245 (34.7)	143 (87.2)
Epidural hematoma, n^a^‡	56 (7.9)	49 (29.8)
Treatment with progesterone, n^a^‡	352 (49.8)	80 (48.7)

*MVC*, motor vehicle collision; *ATV*, all-terrain vehicle, *ED*, emergency department; *GCS*, Glasgow Coma Scale score.

a Parentheses indicate percentage of total

† Indicates difference between the non-operative and operative groups is P<0.05

‡ Indicates difference between the non-operative and operative groups is P<0.001

**Table 3:** Using a threshold of 3 for the SITI (Surgical Intervention for Traumatic Injury) score, the sensitivity, specificity, positive predictive value (PPV), and negative predictive value (NPV) are shown.

	Operative patients	Nonoperative patients		
SITI Score > 3	152	327	PPV	0.39
SITI Score < 3	12	470	NPV	0.97
	Sensitivity	Specificity		
	0.93	0.66		
